# Elevated Gestational IL-13 During Fetal Development Is Associated With Hyperactivity and Inattention in Eight-Year-Old Children

**DOI:** 10.3389/fimmu.2019.01658

**Published:** 2019-07-23

**Authors:** Loreen Thürmann, Gunda Herberth, Ulrike Rolle-Kampczyk, Stefan Röder, Michael Borte, Martin von Bergen, Irina Lehmann, Saskia Trump

**Affiliations:** ^1^Molecular Epidemiology Unit, Charité – Universitátsmedizin Berlin, Corporate Member of Freie Universität Berlin, Humboldt-Universität zu Berlin and Berlin Institute of Health, associated partner of the German Center for Lung Research (DZL), Berlin, Germany; ^2^Department of Environmental Immunology, Helmholtz Centre for Environmental Research-UFZ, Leipzig, Germany; ^3^Department of Molecular Systems Biology, Helmholtz-Centre for Environmental Research-UFZ, Leipzig, Germany; ^4^Children's Hospital, Municipal Hospital “St. Georg”, Academic Teaching Hospital of the University of Leipzig, Leipzig, Germany; ^5^Department of Biochemistry, Faculty of Biosciences, Pharmacy and Psychology, University of Leipzig, Leipzig, Germany

**Keywords:** hyperactivity, inattention, SDQ, maternal, atopic dermatitis, inflammation, prenatal, PUFA

## Abstract

Maternal immune activation (MIA) during fetal development leads to behavioral and psychological disorders in the offspring. Concomitantly, insufficient supply of polyunsaturated fatty acids (PUFAs) is suspected to contribute to early neuronal maldevelopment due to the immune modulatory capabilities of PUFAs. However, human data are missing considering both of these aspects and their impact on children's behavioral outcomes. In line, this study aimed to elucidate the influence of gestational cytokines and PUFA-containing lipids during late pregnancy on behavioral sequelae in childhood, particularly focusing on an immune activation shaped by a history of maternal atopic diseases instead of a pathogen-mediated immune response. Based on the prospective mother-child cohort LINA we assessed the unstimulated blood cytokine profiles and concentrations of PUFA-containing lipids of 293 mothers at the 34th week of pregnancy. Maternal history of atopic diseases was obtained from questionnaires and behavior in eight-year-old children was assessed by the standardized Strength and Difficulties Questionnaires (SDQ) generating scores for hyperactivity/inattention, emotional symptoms, conduct problems, and peer relationship problems. Elevated IL-13 increased the risk for the child to show behavioral difficulties, in particular, hyperactive/inattentive behavior [adj. OR (95% CI): 2.47 (1.51–4.02), *n* = 255 vs. 38] at the age of eight years. Although the presence of maternal atopic dermatitis (AD) was associated with increased gestational IL-13 concentrations [adj. MR (95% CI): 1.17 (1.04–1.32)], no effect on children's behavioral difficulties was observed. However, a decrease in the PUFA containing lipid species PC aa C38:6 was not only associated with an increased gestational IL-13 concentration but also mediated the indirect effect of low PC aa C38:6 concentrations on children's abnormal behavior independent of maternal AD. We additionally assessed whether maternal IL-13 and PC aa C38:6 concentrations translate their effect by altering children's cord blood PC aa C38:6 and IL-13. While also the children's cord blood IL-13 was related to children's behavior, no effect of children's PC aa C38:6 was observed. This is the first study demonstrating that elevated gestational IL-13 increases the risk for children to develop behavioral difficulties. Analyses suggest that a reduced supply of gestational PC aa C38:6 contributes to elevated gestational IL-13 leading to behavioral sequelae in the offspring.

## Introduction

Fetal brain development is highly sensitive to maternal immune activation (MIA). As maternal cytokines, hormones, as well as immune cells can cross the placenta—commonly referred to as “vertical transfer”—changes in the maternal immune status can significantly affect fetal development (1). MIA by infections during pregnancy has been shown to have a profound impact on developing neural circuits and has been linked to the pathogenesis of behavioral and psychological disorders in the offspring (1, 2) like schizophrenia, autism spectrum disorder (ASD), or attention-deficit/hyperactivity disorder (ADHD) (3–5). These associations have been demonstrated in epidemiological studies and were further characterized in rodent models (5–7). Thereby, several lines of evidence suggest that in particular gestational cytokines act as mediators in altered fetal neuroimmune interactions leading to neurobehavioral sequelae in the offspring (8).

Although various insults can trigger changes in the maternal immune homeostasis, best studied are changes in the profile of classical pro-inflammatory cytokines like IL-6, IL-8, or TNFα as a consequence of bacterial or viral infections and their impact on fetal brain development (9–12). Complementary studies in mice support the notion that offspring behavioral abnormalities are attributable to a maternal inflammatory immune response mediated by maternal-fetal cytokine signaling (13). For instance, it has been demonstrated that maternal IL-6 acting via placental IL-6 receptor signaling (14) is responsible for the offspring's development of social and behavioral deficits induced by injection of the viral mimic poly(I:C) into pregnant mice (15).

However, growing evidence suggests that immune activation in the mother also in the absence of pathogens, for instance, due to maternal autoimmune diseases, or an atopic phenotype, may have neuropathological consequences for the child. Recently, Nielsen et al. demonstrated in a Danish nationwide explorative study including 983,680 children that a history of maternal autoimmune diseases such as type 1 diabetes and psoriasis was associated with an increased risk for the offspring to develop ADHD (16). Maternal psoriasis has also been related to an increased risk for ASD in the child (17). In their epidemiological study, Croen et al. could also show that maternal asthma and allergy can contribute to an increased risk for the child to develop ASD (17).

Although adverse effects of MIA during pregnancy have been well described as risk factors for the development of schizophrenia, ASD, or epilepsy in the offspring, there is little information on the contribution of maternal atopic diseases in particular to the highly prevalent neurodevelopmental disorder ADHD characterized by hyperactivity and inattention. In particular, human data investigating the association between gestational cytokine levels and ADHD development are missing.

Further important mediators affecting both the maternal immune status as well as the fetal brain development are poly-unsaturated fatty acids (PUFAs). During pregnancy, high amounts of PUFAs are transported across the placenta to ensure proper brain development, particularly during the last trimester of pregnancy, which is characterized by rapid neuron development and wiring (18, 19). Already Lamptey and Walker could show in a rodent model that a deficiency of PUFAs in the maternal diet led to impaired learning and memory functions in the offspring (20). Moreover, animal studies showed that deficiency of omega-3 fatty acids during gestation and lactation induce a prodromal state of schizophrenia (21), impaired spatial memory (6), and prolonged escape latency in the Morris water maze test (22), while omega-3 fatty acid supplementation improved the performance of the animals (23). On the other hand, due to their immunomodulatory activity gestational PUFA supplementation has beneficial effects in animal models of MIA-induced ASD and schizophrenia. Although in humans benefits of PUFA supplementation during pregnancy are ambiguous (24), in ADHD patients PUFA supplementation might improve symptoms (25).

In the present study, we investigated the potential contribution of maternal atopy-related immune inflammation on behavioral outcomes including hyperactivity/inattention in eight-year-old children of the German population-based prospective mother-child cohort LINA using the standardized Strength and Difficulties Questionnaire (SDQ). The SDQ is an internationally accepted instrument assessing child behavior and has been proven as a valid screening tool for ADHD and hyperactivity/inattention in children (26–30). We investigated the history of maternal allergic diseases such as atopic dermatitis and asthma as potential contributing factors to maternal immune response and the different behavioral difficulty scales of the SDQ. As we not only have information on the maternal cytokine profile during pregnancy but also on the gestational concentrations of different lipid species, we were able to study the interplay between these two immune modulators and their potential impact on the development of behavioral difficulties in children.

## Methods

### Study Cohort

This study is based on the prospective mother-child cohort LINA (Lifestyle and Environmental Factors and their Influence on Newborn's Allergy Risk), for which 629 mother-child pairs were recruited from March 2006 until December 2008 in Leipzig, Germany. Mothers suffering from severe immune or infectious diseases during pregnancy were excluded from the study. Standardized questionnaires (self-administered by the parents) on lifestyle/environmental exposures, living conditions, parental, and children's atopic diseases were recorded during pregnancy and annually after birth. Maternal atopic diseases were defined according to the questions “Did you ever suffer from asthma?,” “Did you ever suffer from atopic dermatitis?” included in the pregnancy questionnaire. Accordingly, the maternal control group for asthma did not report asthma and the control group for AD did not report AD. Information on potential confounding variables was also obtained from this questionnaire. Blood samples were obtained during a clinical investigation of the mother in the 34th week of gestation and for the children at the time of birth (cord blood) and annually after birth at scheduled clinical visits. This study was carried out in accordance with the declaration of Helsinki and was approved by the Institutional Review Board of the University of Leipzig (046-2006, 150/14-ff). Participation in the study was voluntary and informed written consent was given by the parents.

This study is based on a LINA subcohort considering all children, for whom maternal blood samples obtained at the 34th week of gestation and SDQ information at children's age of eight were available (*n* = 293).

### Behavioral Evaluation (SDQ)

Behavioral evaluation of eight-year-old children was based on the standardized Strength and Difficulties Questionnaire (SDQ) assessing emotional, mental, and behavioral problems.

Parents completed the 25-item SDQ questionnaire. Each question could be answered on a three-point scale (0, 1, or 2; “not true,” “somewhat,” or “certainly true”). Five subscores were defined based on five out of the 25 items and corresponded to the following behavioral categories; “hyperactivity/inattention,” “emotional symptoms,” “conduct problems,” “peer relationship problems,” and “prosocial behavior” as developed by Goodman et al. (31). The hyperactivity/inattention subscale is a valid predictor of clinically diagnosed ADHD (32) and is composed of five items (“Restless, overactive, cannot stay still for long”; “Constantly fidgeting or squirming”; “Easily distracted, concentration wanders”; “Thinks things out before acting”; and “Sees tasks through to the end, good attention span”) addressing the three core symptoms of ADHD diagnosis criteria according to the Diagnostic and Statistical Manual of Mental Disorders—Fourth Edition (DSM-IV—American Psychiatric Association, 1994), namely inattention and hyperactivity/impulsiveness behavior (26).

A score for each of these subscales was calculated by summing the scored answers of each of the five items (range 0–10). A total difficulties score (TDS) was obtained by summing up the four subscores “emotional symptoms,” “conduct problems,” “hyperactivity/inattention,” and “peer relationship problems” for each child ranging from 0 to 40. Subsequently, based on the normalization of parent-reported SDQ of the German-wide national study, the children were classified as showing “normal,” “borderline,” or “abnormal” behavior based on Woerner et al. (33). Since the prosocial behavior score is not included in the TDS calculation, we omitted the prosocial subscale in all subsequent analyses (31). For statistical analyses, children with borderline and abnormal behavior were combined.

### Cytokine Measurement

Cord blood and maternal heparinized blood obtained at the 34th week of gestation by venous puncture was used for cytokine measurements. Aliquots of these samples were diluted with RPMI medium and incubated for 4 h at 37°C. After centrifugation, resulting supernatants were used to determine unstimulated cytokine concentrations in [pg/ml] (maternal: IL-4, IL-5, IL-6, IL-8, IL-10, IL-12, IL-13, TNFα, IFNγ, MCP-1; cord blood: IL-13) using the BD CBA Human Soluble Flex Set system (BD Bioscience, Heidelberg, Germany) as previously described (34, 35). Cytokine concentrations below the detection limit were assigned a value that was half of the detection limit prior to ln-transformation.

### Measurement of Blood Lipids

Metabolomic analysis was performed with 10 μl of blood serum (maternal: 34th week of gestation; infantile: cord blood) by using the AbsoluteIDQ p180 Kit (Biocrates Life Science AG, Innsbruck, Austria) as described elsewhere (36). Briefly, in total 180 compounds were measured including sugars, amino acids, acylcarnitines, biogenic amines, and lipids. For lipid analysis, the AbsoluteIDQ p180 Kit employs a flow injection analysis tandem mass spectrometry (FIA-MS/MS) and generates lipid class-specific fragment ions. As such, each of the detected signals is a sum of different isobaric/isomeric lipids. Measured lipid species include phosphatidylcholines (PC), lysophosphatidylcholines (LysoPC), and sphingomyelins (SM). In the current study, we focused on lipids containing polyunsaturated fatty acids (PUFAs). Therefore, only PC lipid species with at least three double bonds, and LysoPC, or SM lipids containing at least two double bonds, were considered to ensure inclusion of lipids containing at least one PUFA. To this end, subsequent analyses were restricted to *n* = 43 PUFA containing lipids. The list of maternal lipid species, which were included in the analyses is provided in **Table 4**.

### Statistical Analysis

Chi-square was used to test for equal parameter distribution in the analyzed subcohort and the entire LINA cohort.

Children's behavioral scores were considered either as continuous variables in multiple regression analyses or as categorical variables in logistic regression analyses. Logistic regression models adjusted for potential confounding variables were applied to study the association of gestational cytokines, cord blood IL-13, maternal atopic diseases, or PC aa C38:6 lipid (gestational and cord blood) on children's behavioral abnormalities. Adjusted multiple regression models were applied for the association analyses of continuous variables such as cytokine concentrations, TDS or hyperactivity/inattentive scores. To evaluate the relationship between PUFA containing lipid species and IL-13 concentrations during pregnancy, Spearman correlations were applied. To study whether maternal IL-13 or cord blood IL-13 mediates the indirect effect of the PUFA containing lipid species “PC aa C38:6” on children's TDS or hyperactivity/inattention, mediation analyses were conducted (PROCESS macro v2.16.3 for SPSS, www.processmacro.org) using a bootstrap approach with 5,000 samples to estimate the statistical significance of the indirect effect as percentile-corrected 95% CIs (37). For multiple regression and mediation analyses, all continuous variables were ln-transformed.

In all models, except those investigating the association between maternal atopic diseases and IL-13 concentration, we considered the potential confounding factors known to influence children's behavior (38–42): gender, parental educational level, household income, tobacco smoke exposure during pregnancy, and alcohol consumption during pregnancy. For multiple regression analyses assessing the influence of maternal atopic diseases on IL-13, the maternal age, the maternal education level, the tobacco smoke exposure during pregnancy, the household income, and cat keeping were included in the models as confounding factors (all derived from questionnaires).

For the comparison of groups, either the Kruskal-Wallis test or Mann-Whitney *U*-test were applied. Whenever applicable we performed multiple test correction by *Bonferroni*. *P* ≤ 0.05 was considered significant. Analyses were performed in either STATISTICA 13.3 for Windows (Dell Inc., USA), GraphPad Prism version 7.00 for Windows (GraphPad Software, USA), SigmaPlot version 14.0 (Systat Software GmbH, Germany), or IBM SPSS Statistics version 25 (IBM Corps., USA).

## Results

### General Study Characteristics and Prevalence of Abnormal Behavior

In total, 334 children participated in the LINA eight-year follow-up investigation. For 293 of these children SDQ information, as well as maternal blood samples collected during the 34th week of pregnancy, were available. General characteristics of the analyzed subcohort (*n* = 293) and the entire LINA cohort (*n* = 629) are given in Table 1. There was no selection bias in the subcohort compared to the total cohort. Overall, 12.6% (*n* = 37) of the children were classified as showing abnormal or borderline behavior based on the German cut-off for the total difficulties score (TDS) (33), which is in line with previous observations reporting that 13.8% of 10 year-old and 13.0% of 6–11-year-old children showed either abnormal, or borderline behavior based on TDS classification (43, 44). According to the standardized classification provided by Woerner et al. (33) for the different SDQ subscores, 13% (*n* = 38) of the children were hyperactive/inattentive, 17.1% (*n* = 50) showed emotional symptoms, 10.2% (*n* = 30) showed conduct problems, and 6.5% (*n* = 19) had difficulties in peer relationships (Table 2).

**Table 1:** Characteristics of the entire LINA cohort and the investigated subcohort.

	**Entire cohort *n* = 629 (%)^a^**	**Subcohort *n* = 293 (%)^a^**	***p*-value^#^**
**Gender of the child**			
Male	330 (52.5)	151 (51.5)	0.848
Female	299 (47.5)	142 (48.5)	
Household income^*^			
Low	139 (22.1)	58 (19.8)	0.230
Intermediate	326 (51.8)	160 (54.6)	
High	125 (19.9)	75 (25.6)	
Tobacco smoke exposure during pregnancy^**^			
(Almost) daily	48 (7.6)	13 (4.4)	0.191
Occasionally	47 (7.5)	22 (7.5)	
Never	534 (84.9)	258 (88.1)	
Parental education level^***^			
Low	16 (2.5)	3 (1.0)	0.066
Intermediate	144 (22.9)	53 (18.1)	
High	469 (74.6)	237 (80.9)	
**Alcohol consumption during pregnancy**			
Never	353 (56.1)	166 (56.7)	0.935
Ever	276 (43.9)	127 (43.3)	
**Maternal history of atopic diseases**	***n*****/*****N*** **(%)**	***n*****/*****N*** **(%)**	
Asthma	71/627 (11.3)	30/293 (10.2)	0.706
Atopic dermatitis	112/623 (18.0)	50/292 (17.1)	0.824

a *Numbers may be different from the total sum due to missing data*.

# *p-value from chi-squared test for cross relationship*.

* *Low income: <1,500€ per month, intermediate income: 1,500–3,000€ per month, high income: >3,000€ per month*.

** Answers based on the question “Did you or anybody else smoke inside your home during the last 12 months?”

*** Low: nine years of schooling or less “Hauptschulabschluss”, intermediate: 10 years of schooling “Mittlere Reife”, high: 12 years of schooling or more “(Fach-)hochschulreife.”

**Table 2:** Prevalence of children with abnormal/borderline behavior at the age of eight.

**SDQ category^a^**	***n*/*N* (%)**
Hyperactivity/inattention	38/293 (13.0)
Emotional symptoms	50/293 (17.1)
Conduct problems	30/293 (10.2)
Peer relationship problems	19/293 (6.5)
TDS*^b^*	37/293 (12.6)

a *Categorization in “normal” and “borderline/abnormal” based on (33)*.

b The score of “total difficulties” is the sum of the categories “hyperactivity/inattention,” “emotional symptoms,” “conduct problems,” and “peer relationship problems.”

### Gestational Cytokine Pattern and Children's Development of Abnormal Behavior

**Figure 1:**
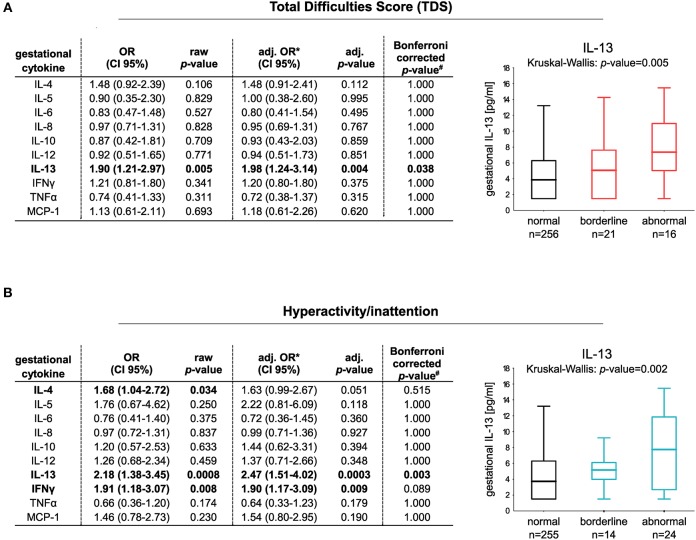
Risk increase for the development of abnormal behavior at the age of eight in relation to gestational cytokine profile. Classification of the children in normal and abnormal behavior is based on **(A)** TDS (256 controls vs. 37 children with abnormal behavior) or **(B)** the hyperactivity/inattention subscore (255 controls vs. 38 children with hyperactive/inattentive behavior). Box plots show gestational IL-13 concentrations of children with normal, borderline, or abnormal behavior (median with 25/75% quartile; whiskers represent the non-outlier range). Differences in numbers are due to the different cut-offs determining normal and abnormal behavior applied for the subscales. Cut-offs were obtained from Woerner et al. (33). Logistic regression models comparing normal vs. abnormal/borderline behavior were adjusted for gender, parental educational level, household income, prenatal tobacco smoke exposure, and alcohol consumption during pregnancy. For analyses, cytokine data were ln-transformed (#*p*-values adjusted for *Bonferroni* correction, *n*(tests) = 10).

We first applied an adjusted logistic regression model to determine whether maternal cytokines (IL-4, IL-5, IL-6, IL-8, IL-10, IL-12, IL-13, MCP-1, TNFα, and IFNγ) measured at the 34th week of pregnancy are linked to the development of abnormal children's behavior in later life. Among the ten cytokines investigated, an increased IL-13 protein concentration was associated with an increased risk for the child to show abnormal behavior at the age of eight based on the TDS [OR (95% CI): 1.90 (1.21–2.97); adj. OR (95% CI): 1.98 (1.24–3.14), *n* = 256 vs. 37, Figure 1A].

Categorizing the children in those showing abnormal/borderline behavior based on the different subscales of the SDQ, it became apparent that increased prenatal-maternal IL-13 exposure is in particular associated with hyperactivity/inattention of children [OR (95% CI): 2.18 (1.38–3.45); adj. OR (95% CI): 2.47 (1.51–4.02), *n* = 255 vs. 38, Figure 1B]. No association of maternal IL-13 to the other subscales of the SDQ was observed (Supplementary Table 1).

We furthermore saw an increased risk for the children to show hyperactive/inattentive behavior at the age of eight in association to elevated gestational IFNγ [OR (95% CI): 1.91 (1.18–3.07); adj. OR (95% CI): 1.90 (1.17–3.09)] and IL-4 concentrations [OR (95% CI): 1.68 (1.04–2.72); adj. OR (95% CI): 1.63 (0.99–2.67)]. However, the association between IL-4/IFNγ/IL-13 and children's hyperactivity/inattention remained statistically significant only for IL-13 following multiple test correction (Figure 1B).

### Association of Maternal Atopic Diseases With Gestational IL-13 and Children's Behavior

**Figure 2:**
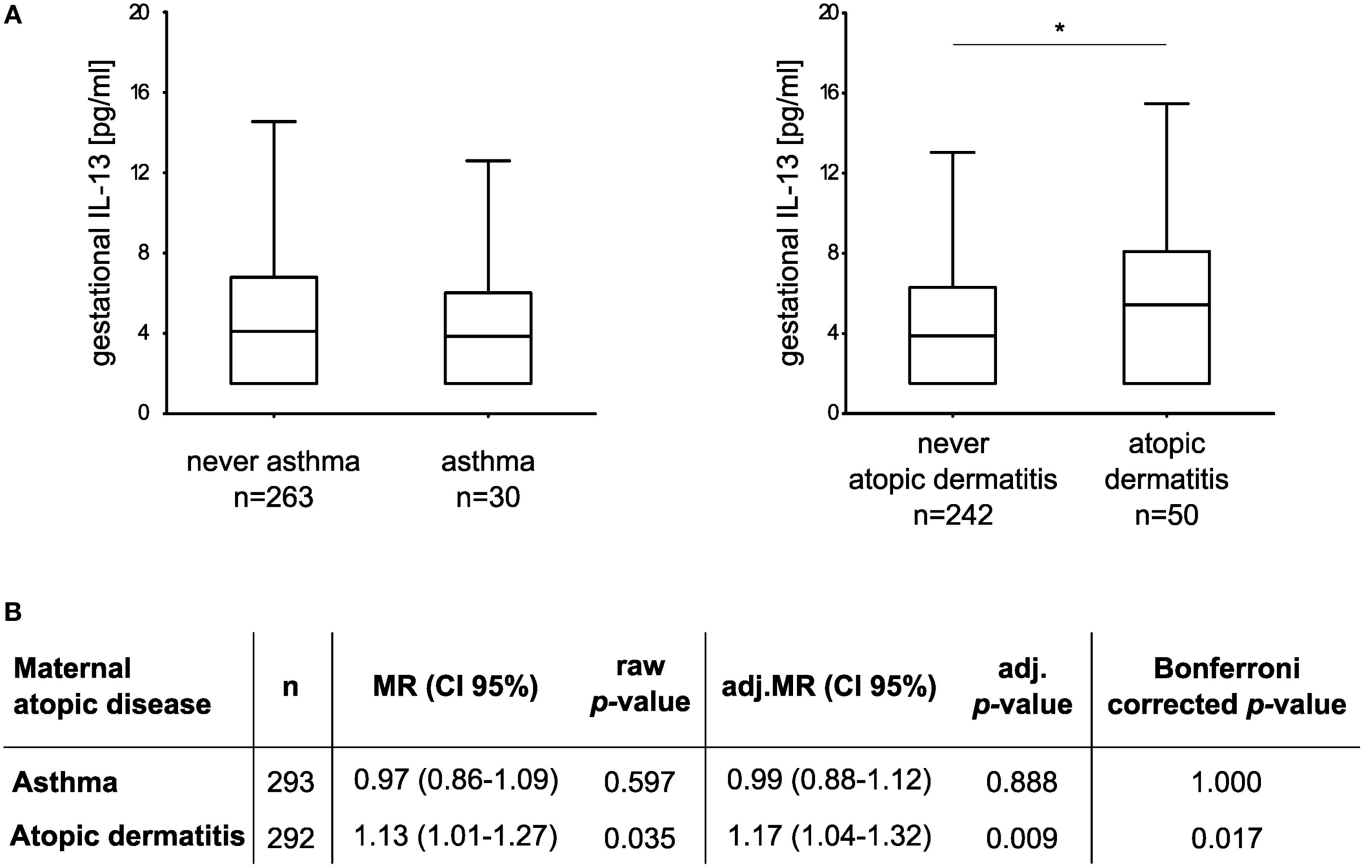
Association between maternal atopic diseases and gestational IL-13. **(A)** Box plots show gestational IL-13 concentrations of mothers suffering from asthma or AD in comparison to their respective controls (median with 25/75% quartile; whiskers represent the non-outlier range, ^*^p < 0.05 from Mann-Whitney *U* test). **(B)** Multiple regression models adjusted for maternal age, maternal educational level, tobacco smoke exposure during pregnancy, household income, and cat keeping. For analyses, IL-13 concentrations (dependent variable) were ln-transformed.

We went on to investigate the influence of maternal atopic diseases, such as asthma, and AD on gestational IL-13 concentrations and the development of behavioral abnormalities in their offspring. The median concentration (and IQR) of gestational IL-13 was 3.85 pg/ml (1.50–6.02 pg/ml) in asthmatic mothers (*n* = 30) and 5.43 pg/ml (1.50–8.09 pg/ml) in mothers suffering of AD (*n* = 50). For asthma controls (*n* = 263) the median concentration was 4.10 pg/ml (1.5–6.80 pg/ml) and 3.88 pg/ml (1.50–6.30 pg/ml) for AD controls, respectively (Figure 2A). Adjusted multiple regression analysis revealed no association of maternal asthma with gestational IL-13. However, a significant influence of maternal AD on increased IL-13 concentration [MR (95% CI): 1.13 (1.01–1.27); adj.MR (95% CI): 1.17 (1.04–1.32), Figure 2B] was observed, which was sustained even after additionally adjusting for asthma [adj.MR (95% CI) = 1.18 (1.04–1.33), *n* = 292, *p* = 0.007].

Children of mothers suffering from AD showed an increased risk for the development of abnormal or borderline behavioral problems at the age of eight only in trend [TDS: adj. OR (95% CI): 2.13 (0.94–4.82), *n* = 255 vs. 37, Table 3], while no association of maternal AD with the development of hyperactive/inattentive behavior was observed. In line, maternal asthma had no impact on children's behavior (Supplementary Table 2).

**Table 3:** Risk increase for children to develop behavioral abnormalities at the age of eight in relation to maternal atopic dermatitis diagnosis.

**SDQ scale**	***n* (control/ abnormal)**	**OR (CI 95%)**	***p*-value**	adj.^*^ OR (CI 95%)	adj.^*^p-value
Hyperactivity/inattention	254/38	1.91 (0.86–4.25)	0.111	1.61 (0.70–3.74)	0.262
Emotional symptoms	242/50	1.08 (0.48–2.39)	0.857	1.05 (0.47–2.37)	0.905
Conduct problems	262/30	0.72 (0.24–2.18)	0.562	0.57 (0.18–1.81)	0.340
Peer relationship problems	273/19	1.81 (0.62–5.30)	0.277	2.01 (0.66–6.12)	0.219
TDS	255/37	2.34 (1.07–5.14)	0.033	2.13 (0.94–4.82)	0.069

* *Logistic regression models were adjusted for gender, parental educational level, household income, prenatal tobacco smoke exposure, and alcohol consumption during pregnancy*.

*The difference in case and control numbers are due to the different cut-offs determining normal and abnormal behavior applied for the subscales. Cut-offs were obtained from Woerner et al. (33)*.

**Figure 3:**
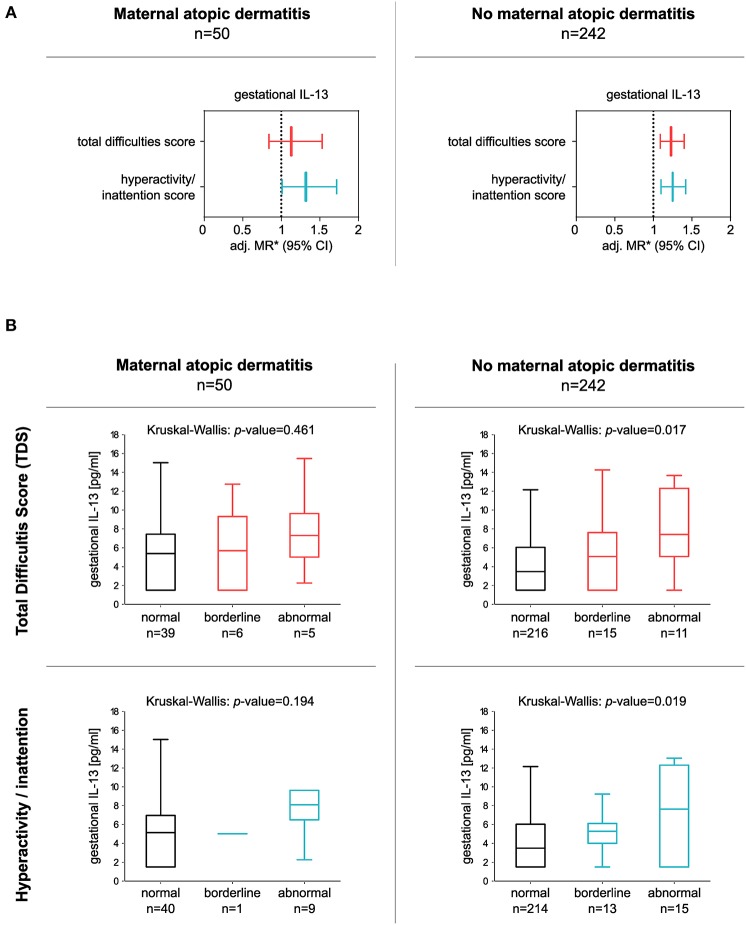
Association between gestational IL-13 and children's TDS or hyperactivity/inattention score in children born to mothers with or without a history of atopic dermatitis (AD). **(A)** Forest plots consider either children of mothers with a history of AD (left panel) or children of mothers without a history of maternal AD (right panel). ^*^MRs from multiple regressions adjusted for gender, parental educational level, household income, tobacco smoke exposure during pregnancy, and alcohol consumption during pregnancy. IL-13 data were ln-transformed. **(B)** Box plots show gestational IL-13 concentrations of children with normal, borderline, or abnormal behavior (median with 25/75% quartile; whiskers represent non-outlier range), for TDS (upper panel), and hyperactivity/inattention subscores (lower panel). Differences in numbers are due to the different cut-offs determining normal and abnormal behavior applied for the subscales. Cut-offs were obtained from Woerner et al. (33).

In addition, the significant association between gestational IL-13 and the hyperactivity/inattention score was still observed in children from mothers with a positive history of AD [adj. MR (95% CI): 1.32 (1.01–1.72), *n* = 50], as well as in children of mothers without AD [hyperactivity/inattention: adj. MR (95% CI): 1.25 (1.10–1.42), *n* = 242]. In the latter, elevated IL-13 concentrations were related to an increase in the TDS as well [TDS: adj. MR (95% CI): 1.23 (1.09–1.40), *n* = 242; Figure 3].

### PUFA Species/Gestational IL-13 and Offspring's Behavioral Difficulties

We went on to investigate 43 PUFA containing lipid species detected in maternal serum at the 34th week of pregnancy as a further potential source of IL-13 increase by applying *Bonferroni* corrected correlation analyses (Table 4). Strikingly, almost half of the phosphatidylcholine lipids (17 out of 38 PC-lipids) were negatively associated with maternal IL-13 while only the lipid species PC aa C38:6 remained statistically significant after multiple testing (Spearman correlation: *R* = −0.21, *Bonferroni* corrected *p* = 0.036, Supplementary Figure S1).

**Table 4:** Correlation between lipid metabolites containing at least one PUFA and gestational IL-13.

**Lipid species containing ≥ 1 PUFA**	**Serum concentration at the 34th week of pregnancy [μM] median (IQR)**	**Spearman correlation**	**Raw *p*-value**	Bonferroni corrected *p*-value^*^
**LYSOPHOSPHATIDYLCHOLINE**				
lysoPC a C18:2	10.78 (8.76–13.51)	0.11	0.0947	1.0000
lysoPC a C20:3	1.52 (1.28–2.04)	0.05	0.4365	1.0000
lysoPC a C20:4	3.31 (2.73–4.59)	0.09	0.1412	1.0000
**PHOSPHATIDYLCHOLINE**				
**PC aa C32:3**	2.24 (1.01–3.07)	−0.15	**0.0211**	0.9083
**PC aa C34:3**	69.42 (38.51–89.07)	−0.16	**0.0102**	0.4396
PC aa C34:4	6.76 (4.07–9.8)	−0.08	0.2273	1.0000
**PC aa C36:3**	361.92 (246.95–424.65)	−0.18	**0.0054**	0.2343
**PC aa C36:4**	458.97 (295.64–560.94)	−0.16	**0.0140**	0.6035
PC aa C36:5	44.84 (29.82–64.59)	−0.10	0.1126	1.0000
PC aa C36:6	3.42 (2.37–4.9)	−0.09	0.1621	1.0000
**PC aa C38:3**	80.45 (64.2–97.07)	−0.15	**0.0185**	0.7939
**PC aa C38:4**	151.22 (119.82–183.03)	−0.13	**0.0474**	1.0000
PC aa C38:5	94.08 (69.37–117.77)	−0.12	0.0632	1.0000
**PC aa C38:6**	**229.62 (175.17–294.09)**	**-0.21**	**0.0008**	**0.0360**
**PC aa C40:3**	1.2 (0.89–1.58)	−0.15	**0.0233**	1.0000
PC aa C40:4	5.58 (4.67–6.87)	−0.09	0.1517	1.0000
PC aa C40:5	14.16 (11.66–17.68)	−0.08	0.2284	1.0000
**PC aa C40:6**	48.14 (41.58–61.66)	−0.19	**0.0037**	0.1600
PC aa C42:4	0.4 (0.34–0.56)	−0.05	0.4012	1.0000
PC aa C42:5	0.84 (0.72–1.09)	−0.10	0.1371	1.0000
PC aa C42:6	1.02 (0.86–1.25)	−0.05	0.4368	1.0000
**PC ae C34:3**	19.03 (10.75–24.25)	−0.17	**0.0061**	0.2637
PC ae C36:3	22.33 (13.78–28.75)	−0.12	0.0680	1.0000
PC ae C36:4	37.66 (25.43–47.1)	−0.11	0.0735	1.0000
**PC ae C36:5**	20.74 (13.24–26.83)	−0.16	**0.0131**	0.5634
**PC ae C38:3**	10.4 (7.15–13.1)	−0.15	**0.0154**	0.6624
**PC ae C38:4**	26.39 (20.57–32.32)	−0.13	**0.0375**	1.0000
**PC ae C38:5**	31.79 (22.03–37.59)	−0.14	**0.0267**	1.0000
PC ae C38:6	15.51 (10.38–18.89)	−0.11	0.0782	1.0000
PC ae C40:3	2.61 (2–3.3)	−0.11	0.0789	1.0000
PC ae C40:4	4.48 (3.74–5.44)	−0.07	0.3037	1.0000
**PC ae C40:5**	6.7 (5.38–8.31)	−0.13	**0.0394**	1.0000
**PC ae C40:6**	8.88 (7.05–10.94)	−0.13	**0.0362**	1.0000
**PC ae C42:3**	1.86 (1.5–2.2)	−0.17	**0.0071**	0.3037
PC ae C42:4	1.62 (1.4–1.97)	−0.02	0.7468	1.0000
PC ae C42:5	3.54 (3.05–4.11)	−0.08	0.2410	1.0000
PC ae C44:3	0.35 (0.27–0.48)	−0.08	0.2009	1.0000
PC ae C44:4	0.72 (0.57–0.94)	0.07	0.2784	1.0000
PC ae C44:5	2.52 (2.12–3.4)	0.11	0.0756	1.0000
PC ae C44:6	1.68 (1.38–2.04)	0.03	0.6584	1.0000
**SPHINGOMYELIN**				
SM (OH) C22:2	4.59 (3.57–10.94)	0.12	0.0528	1.0000
**SM C20:2**	0.47 (0.34–0.78)	0.18	**0.0046**	0.1974
SM C22:3	0.53 (0.26–1.35)	0.10	0.1284	1.0000

* *p-value adjusted for Bonferroni correction, n (tests) = 43*.

*n = 244, p <0.05 are highlighted in bold. Significant correlation after multiple test correction is indicated in orange*.

Due to the nature of the applied AbsoluteIDQ p180 Kit of Biocrates to measure lipids the exact fatty acid linked to the glycerol backbone cannot be determined. However, the possible isotype of the phosphatidylcholine lipid PC aa C38:6 according to the LIPID MAPS structure database (45) the vast majority (18 out of 25 lipids) of the possible PUFAs are omega-3 and/or omega-6 fatty acids including docosahexaenoic acid [DHA, 22:6(n-3)] and eicosapentaenoic acid [EPA, 20:5(n-3), Supplementary Figure S2].

**Figure 4:**
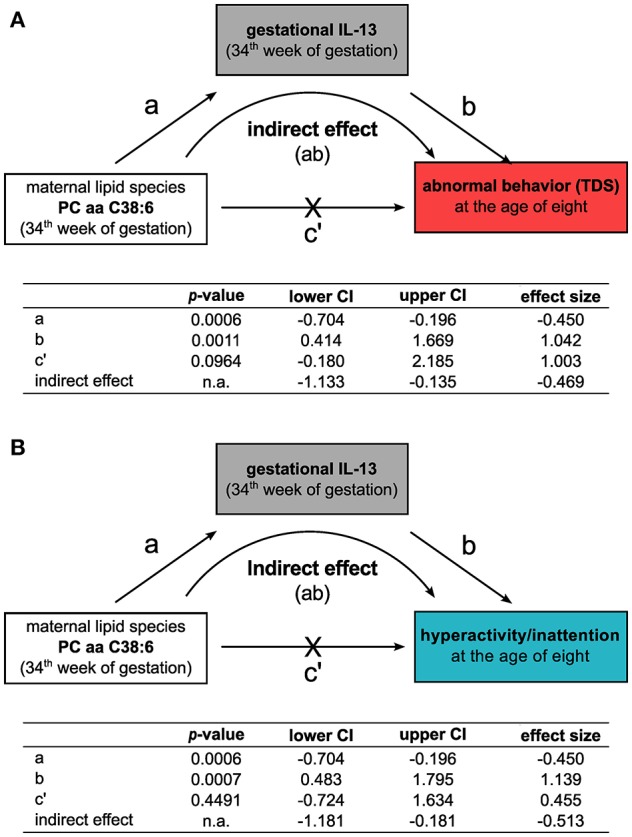
Gestational IL-13 mediates the indirect influence of the maternal phosphatidylcholine lipid species PC aa C38:6 on the development of behavioral difficulties. Mediation analyses for the relationship of the maternal phosphatidylcholine lipid PC aa C38:6, gestational IL-13 and their impact on the development of abnormal behavior at the age of eight based on **(A)** TDS or **(B)** hyperactive/inattentive behavior. Tables summarize unstandardized effect sizes (*n* = 243, adjusted for SDQ confounders and for the history of maternal AD, significance determined by percentile-corrected 95% CI of 5,000 bootstrapped samples, n.a.: no *p*-values are calculated for this model type by the PROCESS macro, effects are significant if confidence intervals do not contain zero).

As PC aa C38:6 showed no direct association to the TDS or to any of the other subscales (Supplementary Table 3), we performed a mediation analysis to evaluate the relationship between maternal PC aa C38:6, IL-13, and children's behavior at the age of eight. The models were adjusted for maternal AD in addition to the confounding factors known to contribute to behavioral sequelae (see Methods section). Here, an indirect effect of PC aa C38:6 on children's TDS as well as hyperactive/inattentive behavior was observed, which was based on PC aa C38:6's negative association with gestational IL-13 (TDS: indirect effect size (lower/upper CI):−0.47 (-1.13/−0.14), Figure 4A; hyperactivity/inattention: indirect effect size (lower/upper CI):−0.51 (-1.18/−0.18), Figure 4B).

### Association of IL-13 With PC aa C38:6 and Their Contribution to Behavioral Sequelae

Next, we investigated whether the effect of gestational IL-13 on the behavioral development of the children might be related to changes in cord blood IL-13 and PC aa C38:6.

An elevated cord blood IL-13 concentration was detected in children with abnormal behavior at the age of eight years based on TDS classification compared to controls [adj.OR (95% CI): 1.77 (1.09–2.87), *n* = 193 vs. 27, Supplementary Table 4, for raw data see Supplementary Figure S3A]. However, in contrast to gestational IL-13 no differential expression was detected in hyperactive/inattentive children compared to controls [adj. OR (95% CI): 1.23 (0.80–1.89), *n* = 191 vs. 29, Supplementary Table 4, for raw data see Supplementary Figure S3B]. In line with maternal PC aa C38:6, cord blood PC aa C38:6 is not altered in children who later develop behavioral sequelae compared to normally behaving children [TDS: adj.OR (95% CI): 0.96 (0.40–2.31), *n* = 32 vs. 203; hyperactivity/inattention: adj.OR (95% CI): 0.84 (0.35–2.01), *n* = 34 vs. 201; Supplementary Table 5].

**Figure 5:**
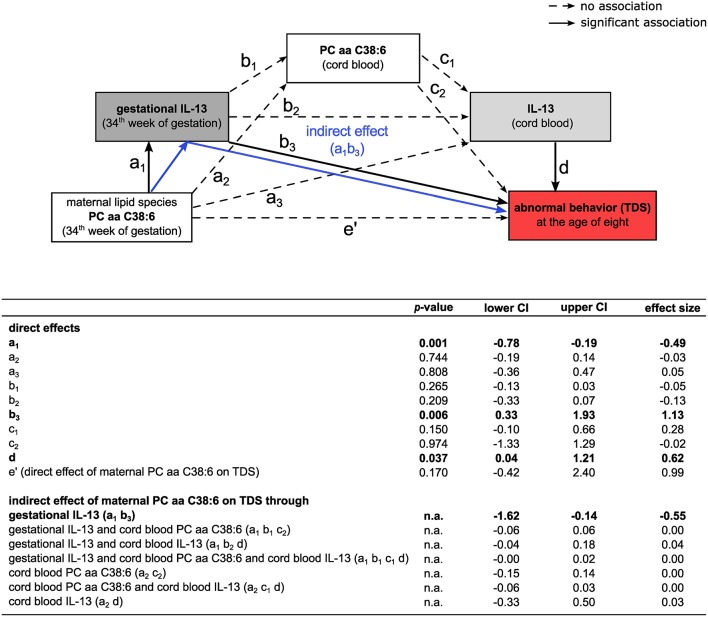
Influence of lipid species PC aa C38:6 and IL-13 on the development of abnormal behavior based on TDS. Mediation analysis of PC aa C38:6 and IL-13 and their impact on abnormal behavior (TDS). Tables summarize unstandardized effect sizes (*n* = 177, adjusted for SDQ confounders and for the history of maternal AD, significance determined by percentile-corrected 95% CI of 5,000 bootstrapped samples, n.a.: no *p*-values are calculated for this model type by the PROCESS macro, effects are significant if confidence intervals do not contain zero).

In a subsequent mediation analysis, the mutual influence of gestational and cord blood IL-13 as well as gestational and cord blood PC aa C38:6 concentrations on children's behavior was assessed. Although, both, gestational and cord blood IL-13 were increased in children with abnormal behavior based on the TDS (Figure 1A and Supplementary Figure S3A), gestational IL-13 was neither directly associated with cord blood IL-13 [direct effect size (lower/upper CI): −0.13 (−0.33/0.07)] nor with cord blood PC aa C38:6 [direct effect size (lower/upper CI): −0.05 (−0.13/0.03), Figure 5]. In addition, the indirect effect of gestational PC aa C38:6 on children's behavior was solely mediated by gestational IL-13 [indirect effect size (lower/upper CI): −0.55 (−1.62/−0.14), Figure 5] and not related to changes in either cord blood IL-13 [indirect effect size (lower/upper CI): 0.03 (−0.33/0.50), Figure 5] or cord blood PC aa C38:6 [indirect effect size (lower/upper CI): 0.00 (−0.15/0.14), Figure 5]. Applying the same mediation model to hyperactivity/inattention, only gestational IL-13, but not cord blood IL-13 was associated with children's hyperactive/inattentive behavior (Supplementary Figure S4).

## Discussion

Based on data of the prospective mother-child cohort LINA, this study evaluated the influence of maternal immune activation (MIA) during the prenatal developmental phase on offspring's behavioral sequelae in eight-year-old children using the standardized SDQ. We focused on the effects of the gestational immune status shaped by a history of maternal atopic diseases rather than on a pathogen-mediated immune response and could show a link between increased gestational IL-13 and children's hyperactivity/inattention and the TDS at the age of eight years. Our analyses included possible alterations of the serum lipid profile during late pregnancy, mainly focusing on PUFA containing lipids, as PUFAs are known as critical immune modulators. To our knowledge, this is the first study investigating the interplay of maternal cytokine and lipid profiles during pregnancy and their impact on children's behavior in later life.

In particular, the end of pregnancy marks a time window highly susceptible to maternal immune insults affecting fetal central nervous system (CNS) development associated with neurodevelopmental sequelae in the offspring. Nowadays, it is well accepted that a maternal inflammatory immune status caused by infectious diseases can increase the risk for children's brain-maldevelopment with behavioral consequences. There is some reason to believe that a maternal non-pathogenic immune activation may contribute to the development of abnormal behavior of the child as well. Elevated maternal IFNγ and IL-4 concentrations during pregnancy have previously been detected in mothers who gave birth to children with later ASD development (46). In our study, we also observed a positive association of concentrations of both of these cytokines during late pregnancy with hyperactive/inattentive behavior in eight-year-old children, although these associations did not sustain significance after multiple test correction.

However, we observed a significantly increased risk for hyperactivity and inattention—two core-symptoms of ADHD—for children exposed to high gestational IL-13 concentrations. In recent years, it has become apparent that the role of IL-13 exceeds that of a classical anti-inflammatory cytokine involved in allergy and parasite infections (47). To date, there are indications that IL-13 is a critical modulator in the CNS and cognition (48, 49) and that increased IL-13 concentrations are associated with ASD and hyperactivity/inattention, respectively (50, 51). Mechanisms by which maternal IL-13 influence brain function of the offspring might be alterations in neuron-microglia interaction. IL-13 expression is described in rodent CA1 pyramidal neurons of the hippocampus (52) as well as in microglia (53–55), which are immune cells of the CNS suspected to translate MIA to neurodevelopmental sequelae (56). In microglia, IL-13 exhibits differing functions (53, 55, 57). While it is accepted that IL-13 concentration can increase in the brain as a result of different infectious or inflammatory insults by activation of microglia, the pathophysiological consequences are controversially discussed (53, 55, 57). It has been suggested that elevated IL-13 leads to the death of activated microglia presumably compensating excessive inflammation *in vivo* (53). On the other hand, enhanced IL-13 triggers reactive oxygen species production in microglia through NADPH oxidase activation leading to the degeneration of hippocampal neurons (57). However, whether these effects of IL-13 on microglia-neuron interaction have consequences for cognition and behavior still needs to be determined.

Findings of our study support the notion that multiple factors influence the observed increased gestational IL-13 concentrations. We provide some evidence that one of these factors is maternal AD, which coincided with elevated serum IL-13 of the mothers. AD is a highly prevalent chronic, inflammatory skin disease characterized by an aberrant systemic immune regulation (58). Cytokines play a pivotal role in AD pathophysiology (59). In particular, IL-13 is suspected as a critical mediator in AD-related inflammatory processes (58). In agreement with our results, peripheral blood IL-13 concentrations are consistently described as being elevated in AD patients (60). However, our data suggests that although maternal AD seems to contribute to elevated maternal IL-13, maternal AD itself is not associated with children's TDS or hyperactivity/inattention. Supported by the finding that maternal IL-13 is also linked to children's behavior in the absence of a maternal AD. This result strongly suggested that further factors influencing maternal IL-13 concentrations potentially play a role in the development of children's behavioral abnormalities.

PUFAs have a well-described immune-regulatory function and contribute to cytokine homeostasis. For instance, *n*-3-PUFA exhibit an anti-inflammatory role by inhibiting inflammatory cytokine production possibly via NR1C3, NFκB, or modulating immune cell activity (61). We observed that high concentrations of maternal IL-13 were also associated with a decrease in the PUFA containing phosphatidylcholine PC aa C38:6 measured in blood sera from the 34th week of pregnancy. These results are consistent with a previous study showing decreased cord blood IL-13 following a PUFA-rich fish oil supplementation during pregnancy (62). Furthermore, lower PUFA concentrations have been found in children with ADHD compared to controls (63, 64) supporting a potential relevance of PUFAs for behavioral abnormalities.

Two possible isomers of PC aa C38:6 contain docosahexaenoic acid (DHA) or eicosapentaenoic acid (EPA), two omega 3-fatty acids essential for healthy brain development (18). Several agencies (e.g., Food and Agriculture Organization of the United Nations, EFSA) recommend supplementation with DHA and EPA for pregnant and lactating women to compensate for increased needs of fetal development particularly of the brain (65, 66). However, randomized control trials were not able to show an apparent beneficial effect of omega-3 fatty acid supplementation during pregnancy on offspring's neurodevelopment (67, 68).

Although, we provide some evidence that maternal as well as cord blood IL-13 are associated with behavioral sequelae in children, they both seem to act independently. Gestational IL-13 was not associated with cord blood IL-13, which is in line with previous observations showing that although maternal Th2 cytokines like IL-5 and IL-6 are related to children's cytokine concentrations at a very early age, for IL-13 no clear relationship has been shown (69). The distinct effects of maternal and fetal IL-13 are supported by the fact that in contrast to gestational PC aa C38:6 cord blood PC aa C38:6 are not associated with changes in IL-13 concentration. This observation suggests that the risk increase for behavioral abnormalities by cord blood IL-13 is independent of PC aa C38:6. Notably, only gestational IL-13, but not cord blood IL-13 was linked to an increased risk to develop hyperactivity/inattention. Therefore, although we observe a mutual deleterious effect of maternal and cord blood IL-13 on children's behavior, it seems that in particular gestational PC aa C38:6 and IL-13 might have a profound influence on brain-development with a long-term impact on behavioral sequelae including childhood hyperactivity/inattention.

Although also previous studies did not observe a direct advantageous effect of omega-3 fatty acid supplementation on children's cognitive outcomes, still our data suggest that a balanced lipid composition during pregnancy is important to sustain cytokine homeostasis (70). Even if transient, such deviations in cytokine homeostasis during critical time windows of development can promote an aberrant CNS architecture leading to an altered brain function in later life.

The findings of this study have to be seen in the light of some limitations. An apparent limitation of our study is the applied metabolome analysis not enabling the determination of specific fatty acids. Thus, we were not able to study the role of maternal PUFA concentration in late pregnancy in more detail and with more accuracy. It is also important to point out that the measurement of PUFAs was performed in serum sample, while the cytokine profile was analyzed in plasma. Although, absolute concentrations might differ between these two different matrices, relative behavior of PUFAs and cytokines should not be affected. Of note, the number of children born to mothers without atopic comorbidities was insufficient to restrict our analysis to only those children. Therefore, we cannot exclude the possibility that also other atopic diseases might contribute to an elevation in maternal IL-13. However, when adjusting our regression analysis for asthma the positive association of maternal AD on IL-13 was sustained. We are also aware of the fact that the use of questionnaires for outcome definition provides less reliable results compared to a clinical diagnosis. Nevertheless, since the applied SDQ is a well-accepted tool validated in numerous studies, we are firmly convinced that the results of this study can add substantial new knowledge to the field (29, 30, 71).

For the first time, we identified increased gestational and cord blood IL-13 as risk factors for the development of behavioral difficulties at a later age. Given the fact that IL-13 emerged as a promising target for neurodevelopmental diseases therapy and is already a therapeutic target for allergy treatment (47), a better understanding of maternal-fetal IL-13 signaling in early brain development is warranted. Therefore, we emphasize to include IL-13 in future studies on MIA mediated psychopathological sequelae of the offspring and to consider pro- and anti-inflammatory cytokines and detailed fatty acid composition in the analyses to investigate whether the presented findings can be validated in independent studies.

## Data Availability

The datasets for this manuscript regarding the LINA cohort generated and/or analyzed in the current study are not publicly available due to limited consent of the study participants but are available from the corresponding author upon reasonable request.

## Ethics Statement

This study was carried out in accordance with the declaration of Helsinki and was approved by the Institutional Review Board of the University of Leipzig (046-2006, 150/14-ff). Participation in the study was voluntary and informed written consent was given by the parents.

## Author Contributions

MB, SR, GH, and IL designed the LINA study, were responsible for the fieldwork and provided proband materials. GH, UR-K, MvB, and IL performed and/or coordinated cytokine and metabolomics measurements. LT and ST performed data analysis. LT, IL, and ST prepared the initial manuscript and figures. All authors contributed to the final manuscript.

### Conflict of Interest Statement

The authors declare that the research was conducted in the absence of any commercial or financial relationships that could be construed as a potential conflict of interest.
