# Supplementary material for: Elevated Gestational IL-13 During Fetal Development Is Associated With Hyperactivity and Inattention in Eight-Year-Old Children
Source: Front Immunol. 2019 Jul 23;10:1658. doi: 10.3389/fimmu.2019.01658 (PMC6690004; doi:10.3389/fimmu.2019.01658)
Supplement: Supplementary file 1 [file Data_Sheet_1.docx]

Supplementary Material

# Supplementary Tables

Supplementary Table 1: Risk increase for the development of behavioral abnormalities at the age of eight in relation to gestational cytokine profile. Note, the difference in case and control numbers are due to the different cut-offs determining normal and abnormal behavior applied for the subscales. Cut-offs were obtained from Woerner *et al.* (Woerner et al. 2002). Cytokine data were ln-transformed.

A) Emotional symptoms (243 controls vs. 50 children with emotional symptoms):

| **gestational cytokine** | **OR**  **(CI 95%)** | **raw**  ***p*-value** | **adj. OR***  **(CI 95%)** | **adj. *p*-value** | **Bonferroni corrected**  ***p*-value^#^** |
| --- | --- | --- | --- | --- | --- |
| IL-4 | 0.855 (0.57-1.29) | 0.451 | 0.85 (0.56-1.28) | 0.434 | 1.000 |
| IL-5 | 0.46 (0.20-1.04) | 0.062 | 0.47 (0.20-1.07) | 0.072 | 0.715 |
| IL-6 | 0.98 (0.63-1.52) | 0.917 | 0.97 (0.62-1.52) | 0.882 | 1.000 |
| IL-8 | 1.00 (0.76-1.31) | 0.975 | 0.99 (0.75-1.30) | 0.923 | 1.000 |
| IL-10 | 0.61 (0.32-1.15) | 0.126 | 0.61 (0.32-1.17) | 0.137 | 1.000 |
| IL-12 | 0.76 (0.45-1.26) | 0.281 | 0.96 (0.67-1.36) | 0.811 | 1.000 |
| IL-13 | 1.01 (0.69-1.49) | 0.943 | 1.03 (0.70-1.51) | 0.893 | 1.000 |
| IFNγ | 0.83 (0.60-1.14) | 0.247 | 0.83 (0.60-1.14) | 0.250 | 1.000 |
| TNFα | 0.95 (0.58-1.55) | 0.836 | 0.94 (0.57-1.55) | 0.816 | 1.000 |
| MCP-1 | 0.86 (0.51-1.46) | 0.576 | 0.83 (0.48-1.44) | 0.506 | 1.000 |

B) Conduct problems (263 controls vs. 30 children with conduct problems):

| **gestational cytokine** | **OR**  **(CI 95%)** | **raw**  ***p*-value** | **adj. OR***  **(CI 95%)** | **adj. *p*-value** | **Bonferroni corrected**  ***p*-value^#^** |
| --- | --- | --- | --- | --- | --- |
| IL-4 | 1.02 (0.61-1.69) | 0.952 | 1.02 (0.61-1.72) | 0.930 | 1.000 |
| IL-5 | 0.62 (0.22-1.71) | 0.352 | 0.70 (0.25-1.97) | 0.498 | 1.000 |
| IL-6 | 0.99 (0.58-1.70) | 0.984 | 1.01 (0.54-1.89) | 0.974 | 1.000 |
| IL-8 | 0.96 (0.69-1.34) | 0.805 | 0.96 (0.67-1.36) | 0.811 | 1.000 |
| IL-10 | 0.93 (0.41-2.08) | 0.854 | 1.02 (0.43-2.45) | 0.963 | 1.000 |
| IL-12 | 0.72 (0.39-1.34) | 0.297 | 0.78 (0.41-1.49) | 0.449 | 1.000 |
| IL-13 | 1.38 (0.86-2.22) | 0.185 | 1.46 (0.90-2.36) | 0.125 | 1.000 |
| IFNγ | 1.06 (0.70-1.61) | 0.793 | 1.05 (0.69-1.62) | 0.809 | 1.000 |
| TNFα | 0.87 (0.47-1.63) | 0.663 | 0.91 (0.46-1.81) | 0.786 | 1.000 |
| MCP-1 | 1.31 (0.66-2.60) | 0.442 | 1.36 (0.66-2.81) | 0.400 | 1.000 |

C) Peer relationship problems (274 controls vs. 19 children with peer problems):

| **gestational cytokine** | **OR**  **(CI 95%)** | **raw**  ***p*-value** | **adj. OR***  **(CI 95%)** | **adj. *p*-value** | **Bonferroni corrected**  ***p*-value^#^** |
| --- | --- | --- | --- | --- | --- |
| IL-4 | 1.51 (0.79-2.87) | 0.210 | 1.51 (0.78-2.93) | 0.218 | 1.000 |
| IL-5 | 1.72 (0.46-6.44) | 0.422 | 1.89 (0.49-7.27) | 0.351 | 1.000 |
| IL-6 | 0.81 (0.36-1.81) | 0.601 | 0.76 (0.33-1.75) | 0.512 | 1.000 |
| IL-8 | 1.05 (0.69-1.59) | 0.831 | 1.04 (0.69-1.58) | 0.844 | 1.000 |
| IL-10 | 1.01 (0.37-2.76) | 0.982 | 0.99 (0.34-2.89) | 0.980 | 1.000 |
| IL-12 | 0.96 (0.43-2.13) | 0.915 | 0.96 (0.42-2.21) | 0.923 | 1.000 |
| IL-13 | 1.42 (0.79-2.55) | 0.240 | 1.40 (0.77-2.54) | 0.268 | 1.000 |
| IFNγ | 1.19 (0.70-2.04) | 0.516 | 1.18 (0.68-2.04) | 0.550 | 1.000 |
| TNFα | 0.87 (0.40-1.88) | 0.728 | 0.83 (0.37-1.89) | 0.659 | 1.000 |
| MCP-1 | 0.84 (0.38-1.85) | 0.665 | 0.84 (0.38-1.88) | 0.670 | 1.000 |

* Logistic regression models were adjusted for gender, parental educational level, household income, prenatal tobacco smoke

exposure, and alcohol consumption during pregnancy.

# *p*-value adjusted for Bonferroni correction, n(tests)=10

Supplementary Table 2: Risk increase for children to develop behavioral abnormalities at the age of eight in relation to maternal asthma. Note, the difference in case and control numbers are due to the different cut-offs determining normal and abnormal behavior applied for the subscales. Cut-offs were obtained from Woerner *et al.* (Woerner et al. 2002).

| **SDQ scale** | **n (control/ abnormal)** | **OR**  **(CI 95%)** | ***p*-value** |  | **adj.* OR**  **(CI 95%)** | **adj.* *p‑*value** |
| --- | --- | --- | --- | --- | --- | --- |
| hyperactivity/inattention | 255/38 | 1.39 (0.50-3.91) | 0.526 |  | 1.31 (0.44-3.90) | 0.622 |
| emotional symptoms | 243/50 | 1.24 (0.48-3.23) | 0.652 |  | 1.23 (0.46-3.28) | 0.674 |
| conduct problems | 263/30 | 0.28 (0.04-2.14) | 0.217 |  | 0.24 (0.03-1.91) | 0.175 |
| peer relationship problems | 274/19 | NA | 1.000 |  | NA | 0.219 |
| **TDS** | 256/37 | 0.73 (0.09-5.90) | 0.769 |  | 1.02 (0.32-3.22) | 0.978 |

* Logistic regression models were adjusted for gender, parental educational level, household income, prenatal tobacco smoke exposure, and alcohol consumption during pregnancy.

Supplementary Table 3: Association between the development of behavioral abnormalities at children’s age of eight and gestational PC aa C38:6 lipid concentration. Note, the difference in case and control numbers are due to the different cut-offs determining normal and abnormal behavior applied for the subscales. Cut-offs were obtained from Woerner *et al.* (Woerner et al. 2002).

| **SDQ categories** | **n (control/ abnormal)** | **PC aa C38:6 concentration [µM]**  median (CI 95%) | | **OR (CI 95%) *p*-value** | | **adj. OR*(CI 95%) *p*-value** | |
| --- | --- | --- | --- | --- | --- | --- | --- |
|  |  | **control group** | **abnormal group** |  |  |  |  |
| hyperactivity/inattention | 213/31 | 232.2 (176.8-296.2) | 208.5 (171.1-280.5) | 0.69 (0.26-1.84) | 0.452 | 0.78 (0.27-2.24) | 0.637 |
| emotional symptoms | 200/44 | 223.5 (175.5-292.9) | 250.2 (159.3-301.9) | 1.33 (0.56-3.16) | 0.521 | 1.47 (0.60-3.60) | 0.398 |
| conduct problems | 219/25 | 224.9 (173.1-294.3) | 262.2 (183.2-291.8) | 1.60 (0.53-4.86) | 0.404 | 1.73 (0.55-5.42) | 0.353 |
| peer relationship problems | 228/16 | 229.6 (174.2-294.8) | 223.4 (176.0-274.1) | 0.82 (0.22-3.10) | 0.774 | 0.96 (0.25-3.65) | 0.954 |
| TDS | 213/31 | 229.6 (173.2-295.2) | 230.7 (176.8-291.8) | 1.21 (0.45-3.29) | 0.702 | 1.39 (0.49-3.94) | 0.535 |

* Logistic regression models were adjusted for gender, parental educational level, household income, prenatal tobacco smoke exposure, and alcohol consumption during pregnancy.

Supplementary Table 4: Association between the development of behavioral abnormalities at children’s age of eight and cord blood IL-13 concentration. Note, the difference in case and control numbers are due to the different cut-offs determining normal and abnormal behavior applied for the subscales. Cut-offs were obtained from Woerner *et al.* (Woerner et al. 2002).

| **SDQ categories** | **n (control/ abnormal)** | **OR**  **(CI 95%)** | ***p*-value** | **adj. OR***  **(CI 95%)** | ***p*-value** |
| --- | --- | --- | --- | --- | --- |
| hyperactivity/inattention | 191/29 | 1.27 (0.84-1.90) | 0.251 | 1.23 (0.80-1.89) | 0.349 |
| emotional symptoms | 183/37 | 1.04 (0.73-1.48) | 0.836 | 1.04 (0.72-1.49) | 0.832 |
| conduct problems | 198/22 | 1.21 (0.77-1.91) | 0.403 | 1.14 (0.71-1.82) | 0.588 |
| peer relationship problems | 204/16 | 1.11 (0.66-1.86) | 0.688 | 1.06 (0.61-1.84) | 0.839 |
| **TDS** | **193/27** | **1.80 (1.13-2.87)** | **0.013** | **1.77 (1.09-2.87)** | **0.021** |

* Logistic regression models were adjusted for gender, parental educational level, household income, prenatal tobacco smoke exposure, and alcohol consumption during pregnancy.

Supplementary Table 5: Association between the development of behavioral abnormalities at children’s age of eight and cord blood PC aa C38:6 lipid concentration. Note, the difference in case and control numbers are due to the different cut-offs determining normal and abnormal behavior applied for the subscales. Cut-offs were obtained from Woerner *et al.* (Woerner et al. 2002).

| **SDQ categories** | **n (control/ abnormal)** | **PC aa C38:6 concentration [µM]**  median (CI 95%) | | **OR (CI 95%) *p*-value** | | **adj. OR*(CI 95%) *p*-value** | |
| --- | --- | --- | --- | --- | --- | --- | --- |
|  |  | **control group** | **abnormal group** |  |  |  |  |
| hyperactivity/inattention | 201/34 | 80.0 (59.4-113.8) | 79.0 (59.3-112.9) | 0.89 (0.40-2.00) | 0.776 | 0.84 (0.35-2.01) | 0.688 |
| emotional symptoms | 197/38 | 78.4 (57.5-114.1) | 87.3 (66.4-107.4) | 1.40 (0.63-3.09) | 0.406 | 1.44 (0.65-3.17) | 0.362 |
| conduct problems | 210/25 | 80.3 (60.4-115.9) | 77.4 (56.9-92.3) | 0.59 (0.24-1.47) | 0.256 | 0.56 (0.22-1.47) | 0.237 |
| peer relationship problems | 219/16 | 80.7 (60.0-115.0) | 75.1 (53.8-89.4) | 0.48 (0.16-1.41) | 0.180 | 0.49 (0.17-1.46) | 0.199 |
| TDS | 203/32 | 81.0 (59.6-110.2) | 79.4 (59.2-113.8) | 0.97 (0.42-2.22) | 0.938 | 0.96 (0.40-2.31) | 0.934 |

* Logistic regression models were adjusted for gender, parental educational level, household income, prenatal tobacco smoke exposure, and alcohol consumption during pregnancy.

# Supplementary Figures


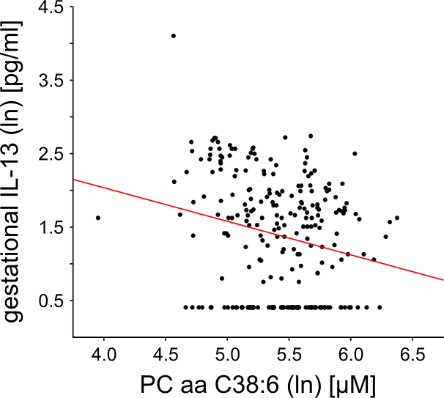


**Supplementary Figure S1: Association between gestational IL-13 and the lipid species PC aa C38:6 concentrations.** Scatterplot represents ln‑transformed data. Spearman correlation: R=-0.21, *p*-value=0.0008, n=244


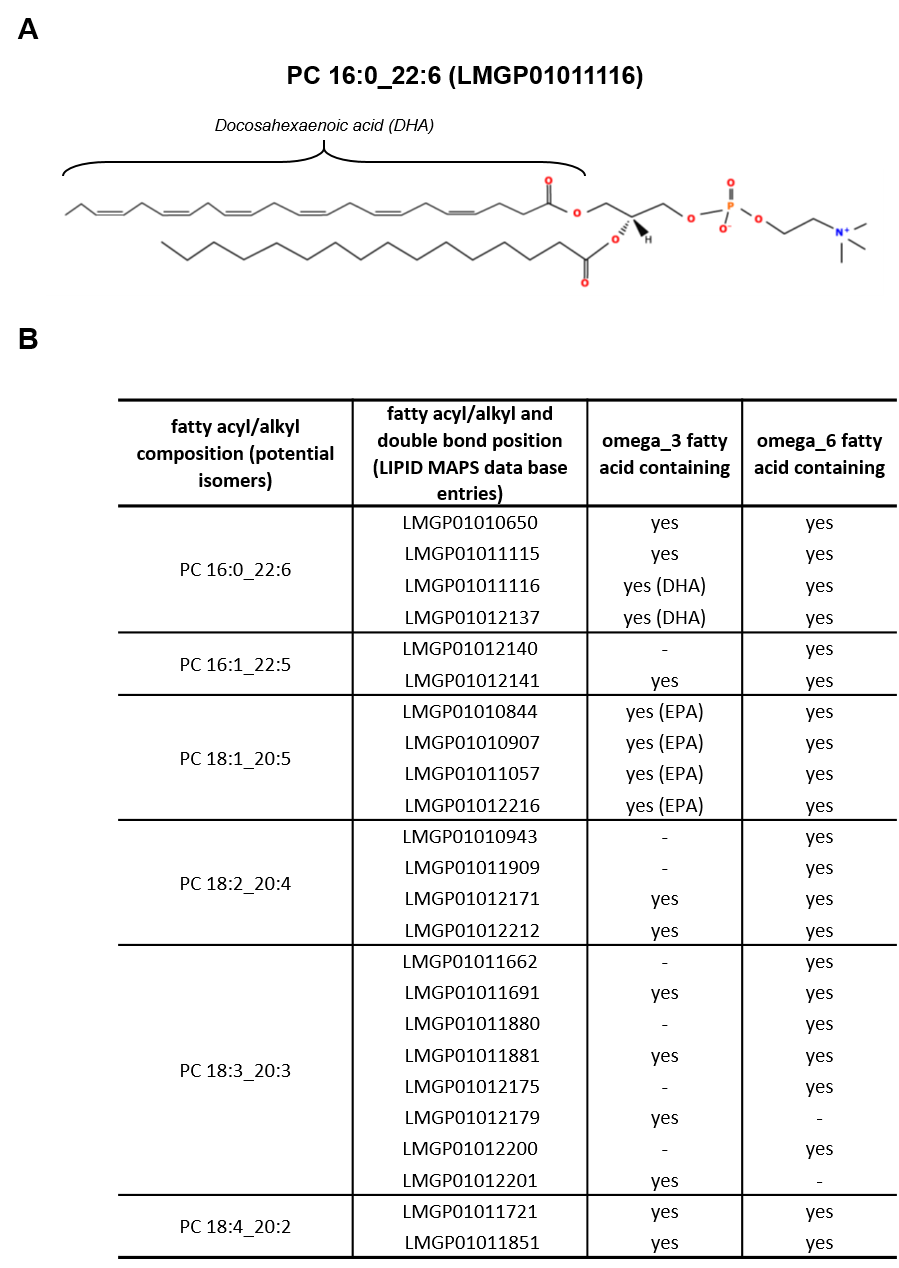


**Supplementary Figure S2.** **Overview of possible PC aa C38:6 isotypes.** **(A)** Chemical structure of the PC aa C38:6 isomer PC 16:0 22:6 containing DHA. **(B)** List of potential lipid isomers of PC aa C38:6 lipid species provided by Biocrates Life Science AG. “aa” = fatty acids are bound via ester bond to the glycerol backbone, “PC” = Phosphatidylcholine, “C#:#” = total carbon number of both fatty acid chains : total number of double bonds

**
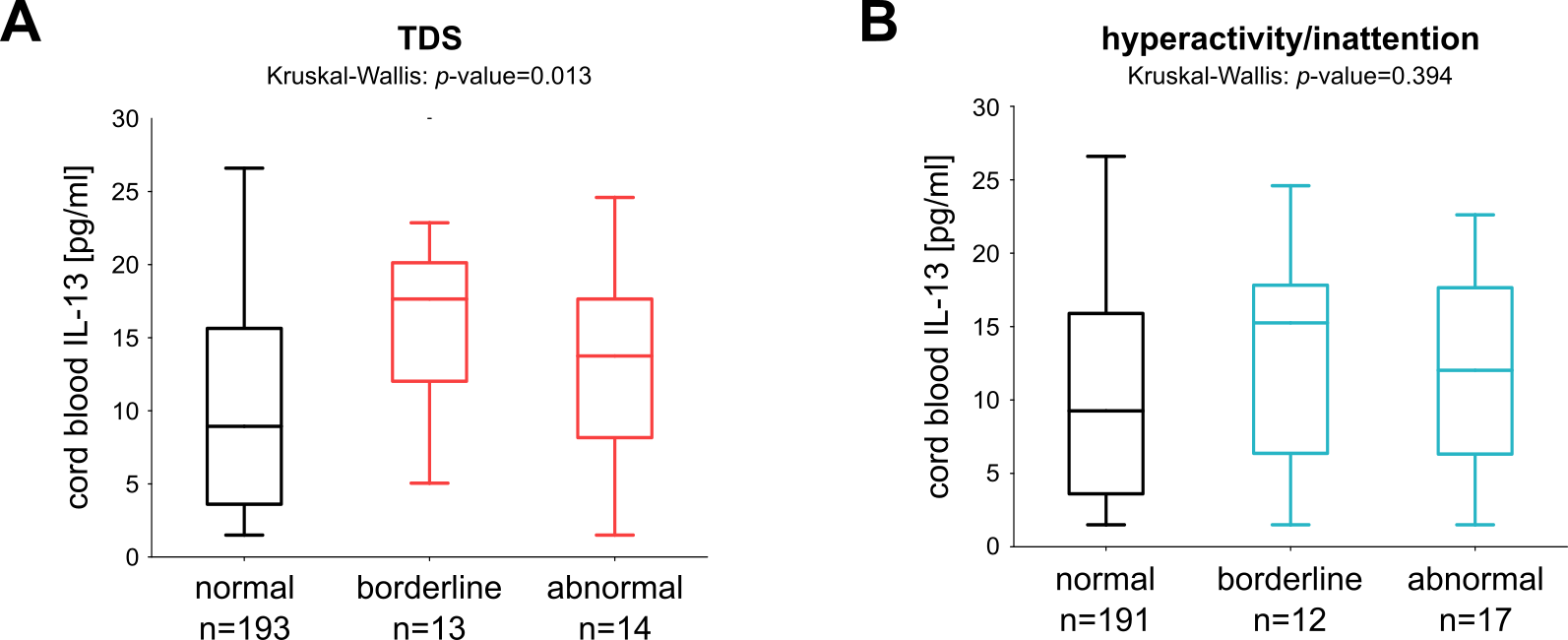
**

**Supplementary Figure S3: Cord blood IL-13 concentrations in children with normal, borderline or abnormal behavior.** Classification of the children in normal and abnormal behavior is based on **A)** TDS or **B)** on the hyperactivity/inattention subscore.

Differences in numbers are due to the different cut-offs determining normal and abnormal behavior applied for the subscales. Cut-offs were obtained from Woerner *et al.* (Woerner et al. 2002). Box plots show cord blood IL-13 concentrations of children with normal, borderline, or abnormal behavior (median with 25/75% quartile; whiskers represent the non-outlier range).

**
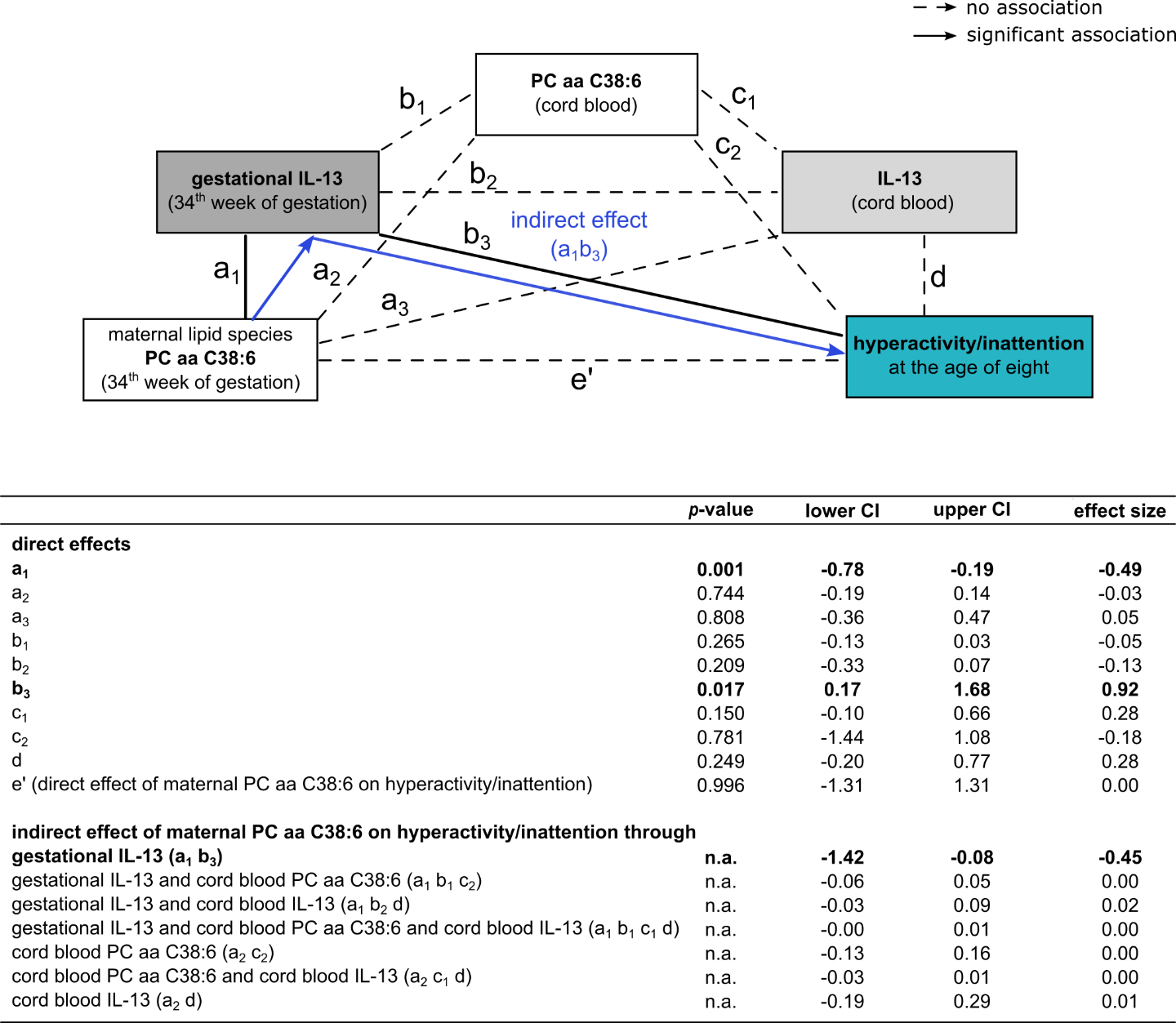
**

**Supplementary Figure S4: Influence of lipid species PC aa C38:6 and IL-13 on the development of hyperactive/inattentive behavior based on hyperactivity/inattention subscore.** Mediation analysis of maternal PC aa C38:6 and IL-13 on the development of hyperactive/inattentive behavior.

Tables summarize unstandardized effect sizes. (n=177, adjusted for SDQ confounders and for the history of maternal AD, significance determined by percentile-corrected 95% CI of 5,000 bootstrapped samples, n.a.: no *p-*values are calculated for this model type by the PROCESS macro, effects are significant if confidence intervals do not contain zero).

**References**

Woerner W, Becker A, Friedrich C, Klasen H, Goodman R, Rothenberger A. 2002. [Normal values and evaluation of the German parents' version of Strengths and DIfficulties Questionnaire (SDQ): Results of a representative field study]. Z Kinder Jugendpsychiatr Psychother 30:105-112.
